# Variants of the hyoid-larynx complex, with implications for forensic science and consequence for the diagnosis of Eagle’s syndrome

**DOI:** 10.1038/s41598-019-52476-z

**Published:** 2019-11-04

**Authors:** Bernadette S. de Bakker, Henri M. de Bakker, Vidija Soerdjbalie-Maikoe, Frederik G. Dikkers

**Affiliations:** 1 Department of Medical Biology, Section Clinical Anatomy & Embryology, Amsterdam UMC, University of Amsterdam, Meibergdreef 9, 1105 AZ Amsterdam, The Netherlands; 2 Department of Radiology, Groene Hart Hospital, Bleulandweg 10, 2803 HH Gouda, The Netherlands; 3 Division of Special Services, Section Forensic Pathology, Netherlands Forensic Institute, P.O. Box 24044, 2490 AA The Hague, The Netherlands; 4 Department of Otorhinolaryngology, Amsterdam UMC, University of Amsterdam, Meibergdreef 9, 1105 AZ Amsterdam, The Netherlands

**Keywords:** Skeleton, Pain

## Abstract

Thorough anatomic knowledge of the hyoid-larynx complex is necessary for forensic radiologists and ear-nose-throat surgeons, given the many anatomic variations that originate in embryology. In forensics the anomalies must be distinguished from fractures because the latter are indicative of violence on the neck. In this manuscript we describe the anatomical variations that can be found in the hyoid-larynx complex and explain their etiology. 284 radiological scans of excised hyoid-larynx complexes were examined with X-ray and CT. Some rare cases from literature and historical collections were added. Two third of the examined hyoid-larynx complexes deviated from the anatomical standard and showed uni- or bilateral ankylosis in the hyoid bone and/or so-called triticeal cartilages. In one fifth of the cases we found striking anatomical variants, mostly derived from the cartilage of the second pharyngeal arch. Anatomical variations of the hyoid-larynx complex can be explained by embryological development. The aberrant hyoid apparatus and the elongated styloid processes (Eagle syndrome) should be considered as one clinical entity with two different expressions as both anomalies are derived from the cartilage of the second pharyngeal arch. Several variants can mimic fractures in this region, so our study is important for radiologists and forensic experts assessing cases of possible violence on the neck.

## Introduction

Anatomical variations of the hyoid-larynx complex occur in 4–30% of the general population^1–4^. Anomalies of this complex are of great importance for radiological examination and surgery of the neck region^1,5^. The significance of anomalies has also been well recognized in forensic sciences, as fractures in this complex are important indicators for strangulation and blunt or penetrating trauma on the neck^5–8^.

The aim of this study was to provide an overview of variations in the hyoid-larynx complex and explain their etiology based on its development. Our data will be discussed in the light of the currently available literature concerning clinical and forensic relevance, providing an overview of this highly polymorphic complex, from development to death.

## Anatomical Variants

### Normal anatomy

**Figure 1:**
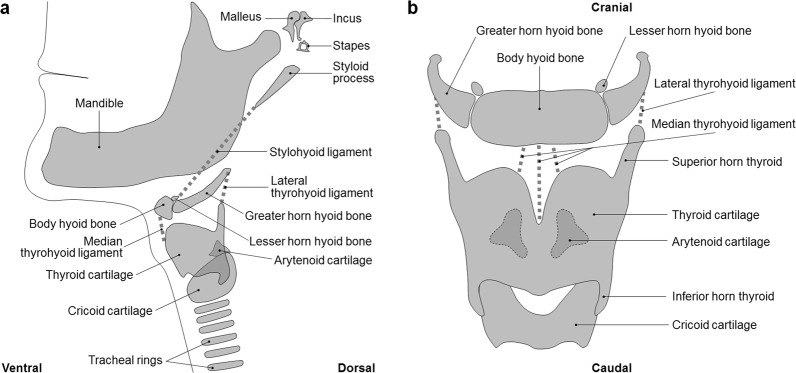
Overview of the normal adult human anatomy of the neck region (**a**). Lateral view of a schematic representation of the normal anatomy of the adult neck region. (**b**) Ventral view of the hyoid bone, thyroid and cricoid region with emphasis on the anatomical structures mentioned in this paper. Note that the arytenoid cartilages (dashed lines) lie in fact dorsally of the thyroid cartilage.

The normal adult hyoid-larynx complex (Fig. 1a) is described as combination of hyoid apparatus (i.e. styloid processes, stylohyoid ligaments and lesser horns of the hyoid), body and greater horns of the hyoid bone, uncalcified thyroid-, cricoid- and arytenoid cartilages and their ligaments proper. The thyroid cartilage encompasses its superior and inferior horns. No additional cartilaginous structures are present in the trajectories of the stylohyoid ligaments, median thyrohyoid ligament and lateral thyrohyoid ligaments and no ankylosis of the joints between hyoid body and greater and/or lesser horns has occurred. Normal length of the styloid process is generally described as 20–30 mm^1,2,9–11^.

### Minor variations

**Figure 2:**
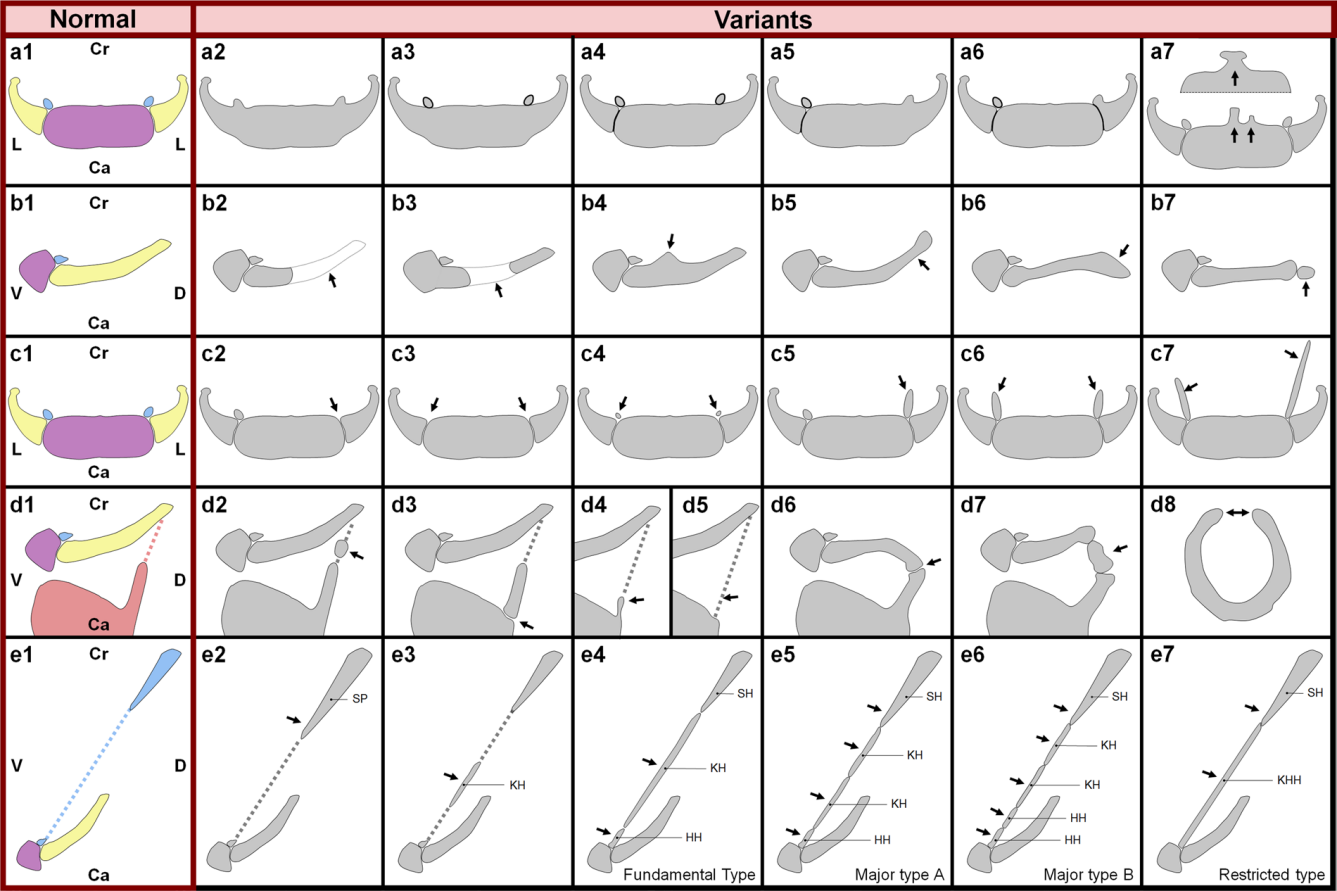
Anatomical variations of the hyoid-larynx complex First vertical column: normal anatomy. The hyoid bone: purple, second pharyngeal arch cartilage derivatives: blue, third pharyngeal arch cartilage derivatives: yellow, thyroid and thyrohyoid ligaments: red. Cr: cranial, Ca: caudal, L: Lateral, V: ventral, D: dorsal. Arrows indicate variation locations. A-row: normal anatomy of the hyoid bone (**a1**) and various degrees of ankylosis in ventral view (**a2**–**a6**). (**a7**) Examples of hyoid bone body exostoses; median process and split median process^12^. B-row: anatomical variations of the greater horn, lateral view. (**b1**) normal anatomy, (**b2**) Hypoplastic, (**b3**) Intermittent, note ankylosis between body and greater horn, (**b4**) Exostosis, (**b5**) Curving upward, (**b6**) Curving downward, (**b7**) Accessory bone. C-row: anatomical variations of the lesser horn, ventral view. (**c1**) Normal anatomy, (**c2**) Unilateral absence, (**c3**) Bilateral absence, (**c4**) Hypoplastic, (**c5**) Unilateral hyperplastic, (**c6**) Bilateral hyperplastic, (**c7**) Asymmetrical hyperplastic. (**d2**–**6**) show the anatomical variations of the thyrohyoid membrane and body of the hyoid bone, lateral view. (**d1**) Normal anatomy, (**d2**) Triticeal cartilage, (**d3**) Non fusion of the superior horn of the thyroid to the thyroid cartilage, this could easily be mistaken for a fracture. (**d4**) Unilateral hypoplastic superior horn of the thyroid cartilage. (**d5**) Uni- or bilateral (P van Driessche, personal communication) absence of the superior horn of the thyroid cartilage. (**d6**) Articulating connection between greater horn and superior horn of the thyroid cartilage. (**d7**) The same as in (**D6**) but with a triticeal cartilage interposed between the two horns. (**d8**) Rare case with a nearly circumferential ankylosed hyoid bone (caudal view)^68^. E-row: lateral view on variations of stylohyoid complex and stylohyoid ligament^36^. (**e1**) Normal anatomy, (**e2**) Elongation of the styloid process (SP); Eagle’s syndrome. (**e3**) A keratohyal (KH) bone in the stylohyoid ligament. (**e4**) Fundamental type with three bones (stylohyal (SH), keratohyal (KH) and hypohyal (HH)). (**e5**) Major type A (stylohyal, keratohyal, keratohyal and hypohyal). (**e6**) Major type B (stylohyal, keratohyal, keratohyal, hypohyal and hypohyal). (**e7**) Restricted type with a fused keratohyal and hypohyal bone, the so called keratohypohyal (KHH) bone.

Minor variations in hyoid-larynx comprise age-related fusion of the body with greater and/or lesser horns by ankyloses of the joints (Fig. 2a2–6), age-related calcification of the thyroid and presence of a triticeal cartilage in the lateral thyrohyoid ligament (Fig. 2d2)^12–16^. Morphological variations of the hyoid are closely related to sex^5,7,17,18^, race^5,6,17^, body proportions^7,19^ and age^5,7,13,14,18,20–24^. European hyoids are broader and shorter than African ones^17^. Distal ends of the greater horns are significantly longer in women than in men^5,18,25^, whereas male hyoids are generally larger than female ones^18,26–28^. Inward curving and flattening of the greater horns are typical for the male hyoids^7^. Furthermore, male hyoids are more susceptible to age modifications^7^. Besides that, males show a higher degree of thyroid ossification, ultimately leading to the completely ossified os thyroideum^12,29^. Finally, hyoid muscle attachment sites also show some individual variation. These minor variations occur so often that they cannot be considered as anatomical variants^14^.

### Age-related ankylosis

Recently, hyoid bone density and ankyloses of the joints between hyoid body and greater and/or lesser horns are getting more attention as possible predictor for age and sex in victim identification^3,30^. Age-related ankylosis (Fig. 2a2–6) is a physiological process that increases with age^5,13,14,18,20–24^. D’Souza reported a mean age of unilateral (Fig. 2a4–5) and bilateral (Fig. 2a2–3) fusion in males of 39.9 and 41.77 years respectively and in females of 37.5 and 45 years^20^. Body and greater horns usually do not fuse until the 35^th^ to 45^th^ year^23,31^ and they might even never fuse^22,23^. Fusion was not reported before the age of 18^32^ or 20^20,30^. Non-fusion (Fig. 2a1) or unilateral fusion (Fig. 2a4–5) has been found in people after the age of 60, which makes this process highly polymorphic^18,20^. Miller *et al*. suggested that fusion is not a continuous ageing process, but that genetic predisposition is the driving force behind this process^18^. Furthermore, sex seems to be of no importance to the fusion process^18,20^. Therefore fusion of the hyoid body with the greater horns cannot be used as an indicator for age or sex^20^.

## Pharyngeal Arch Cartilage Anomalies

Significant anatomical variants are due to the persistence of embryological cartilage^33^. One example is the complete ossification of the lateral thyrohyoid ligament between greater horns of the hyoid bone and superior horns of the thyroid cartilage, called the *congenital hyothyroid bar* (Fig. 2d6–7)^23,33^. Other cases comprise anomalies of the second pharyngeal arch cartilage, such as *stylohyoid syndrome* (*Eagle’s syndrome*)(Fig. 2e2) and the *aberrant hyoid apparatus* (Fig. 2e3–7). The exact incidence of anatomical changes in the stylohyoid chain is difficult to determine, since Eagle’s syndrome and the aberrant hyoid apparatus are often intermingled in literature^1^. It seems to vary from 4% or 5%^1,2,4^ to 28% or 30%^2,34^. Less than 10% of the patients in this group displays clinical symptoms^1,2,4,35^.

### Aberrant hyoid apparatus

Dwight stated in 1907 that Marchetti of Padua’s report from 1652 was the earliest reference to the aberrant hyoid apparatus^33^, also known as (incompletely or completely) ossified hyoid apparatus^23,36,37^. Reichert noted the anatomical connection between styloid process and hyoid in 1837, and assigned its origin to the second pharyngeal arch cartilage^38^. The hyoid apparatus consists of styloid process, stylohyoid ligament and lesser horn of the hyoid^1,11,23,37^. This chain is completely derived from the second pharyngeal arch cartilage (Reichert’s) and can be subdivided into five, or even seven^11^, osteocartilaginous elements from the base of the skull to the hyoid (Table 1)^1,4,10,11,33,37,39–42^. In 1923 Olivier designated the (partly) ossified hyoid apparatus into three main types, depending on the number of bones in the trajectory of the hyoid apparatus (Table 1)^36^.

**Table 1:** Terminology concerning the (ossified) hyoid apparatus, as described by Olivier.

**Terminology concerning the hyoid apparatus from cranial to caudal**			
Tympanohyal	Intrapetrosic part of the styloid process		
Stylohyal	Styloid process		
Keratohyal	Stylohyoid ligament		
Acessory Keratohyal	Stylohyoid ligament		
Hypohyal	Lesser horn hyoid bone		
**Three types of ossified hyoid apparatus described by Olivier**		**Fig**.	**% cases**
The fundamental type with 3 bones > stylohyal, keratohyal and hypohyal		2e4	64%
The mayor type A with 4 bones > stylohyal, keratohyal, accessory keratohyal and hypohyal		2e5	12%
The mayor type B with 5 bones > stylohyal, keratohyal, accessory keratohyal, accessory hypohyal and hypohyal		2e6	
The restricted type with 2 bones > stylohyal and the keratohypohyal (=fused keratohyals and hypohyal)		2e7	24%

Partial ossification of the hyoid apparatus is not uncommon but the appearance of a complete bony chain is rare in humans^37^. This condition is usually bilateral where both sides can differ in symmetry^33^, but it also occurs unilaterally^23,33,37^. This chain passes between internal and external carotid arteries^33^. There is usually some movement possible by a joint or a ligamentous connection between different parts of the chain or at least between the ossified chain and hyoid body^33^.

The aberant hyoid apparatus is hypothesized to originate from persisting second pharyngeal arch cartilage that continued to grow and gradually ossified into a bony chain^11,33^. The joints in the chain often show some degree of bone clubbing, which implies a continuation of growth^33^.

Symptoms include difficulty in swallowing^33^ and restriction of neck movement^36^, but they rarely occur before the age of 40 because of the age dependent ossification of the cartilaginous bar^37^. However, striking examples have also been seen in children^23^. Associated compressive pathologies^1^, like glossopharyngeal neuralgia^43^ or referred pain due to irritation of the sensory nerve branches^44^ have been noted. Also, arterial anomalies in the affected region are not uncommon^37^.

### Eagle’s syndrome

The stylohyoid syndrome, or Eagle’s syndrome (Fig. 2e2), describes a collection of clinical symptoms related to anomalies in size and location of the styloid process, which disturbs surrounding anatomical structures^1,2,4,9,10,23,34,41,45–47^. This condition may be uni- or bilateral and varies in severity^9,10^. The styloid process consists of the tympanohyal and stylohyal part (Table 1)^10,33^. This cylindrical, needle shaped bone, with a cartilaginous tip that normally lies between the internal and external carotid artery, projects ventrocaudally from the inferior side of the petrous bone^1,9,10^. It provides an anchorage for the stylopharyngeus, stylohyoid and styloglossus muscles^1^. During normal development, the cranial part of the second pharyngeal arch cartilage ossifies and forms the styloid process, which is connected to the lesser horn of the hyoid through the stylohyoid ligament^38^. There is no agreement on normal length of the styloid process^34^. It is described as 20–30 mm^1,2,9–11,46^, 30–35 mm^34^ or even 45 mm^48^. The length might be age dependent since an elongated process is more often observed in patients of 30 years and older^1,2,9^, though it has recently also been described in a 9-year-old boy^49^.

The otolaryngologist Watt Eagle described two clinical presentations of stylohyoid syndrome^9,10,46,47^. First the more common *classic type*, which is characterized by foreign body sensation in the throat^2,9,34,41,46^ and dysphagia^2,34,41^. The recurrent dull and not sharply localized facial and cervical pain^2,4,9,23,34,45,46^, radiates towards temporo-mandibular joint^34,47^, mandible^34^, maxillar or mandibular teeth^34^, ear^1,2,23,34,46^, mastoid region^2^, neck^1,34^, tongue^1^, and throat. Pain usually increases toward the end of the day, with turning of the head and after long speaking or singing^34,50^. Eagle also included all cases with distortion of nerve function by the elongated styloid process, involving sensory and motor fibers of the 5^th^, 7^th^, 9^th^ and 10^th^ cranial nerves^41,46^. Patients can suffer from increased salivation^2,46^, a distorted sensation of taste^46^, esophageal and pharyngeal spasms and recurrent coughing^46,51^. The above described symptoms generally occur immediately after tonsillectomy and Eagle believed that the cause of the symptoms was the scar tissue formation, stretching the nerve endings^4,9,46^.

In the second clinical presentation of stylohyoid syndrome, the *stylo-carotid syndrome*, symptoms are found along the distribution of the internal or external carotid artery, due to impingement on the vessel. The styloid process affects the circulation of the carotid arteries and induces irritation of sympathetic nerves in their arterial sheaths^9,41^. With an affected internal carotid artery, patients will complain of parietal headaches and pains in the distribution area of the ophthalmic artery^4,9,41,46^. The elongated styloid process can push the internal carotid artery laterally, which may be painful on palpation^46^. When the external carotid artery is affected, pain will be referred to the temple and infraorbital region^9,41^. There is even a hypothesis that tinnitus can be caused by stylo-carotid syndrome. This could be explained because pulsating waves from the artery are conducted through the elongated styloid process towards skull and cochlea^41,46^.

A vegetative syndrome, including pallor, sweating, hypotension and even brief loss-of-consciousness episodes, due to irritation of the carotid perivascular plexus or carotid body by the elongated styloid process, has also been reported^1,2,41,52^. Wilmoth and Leger described this phenomenon as ‘*Syncope styloidea*’, which occurred in patients with a combination of a high bifurcation of the common carotid artery and an elongated styloid process^41^.

The elongated styloid process can be palpated in the tonsillar fossa during clinical examination^2,46^. For radiological imaging, the computed tomography (CT) preferably with 3D reconstruction is the modality of choice^2,4^. Sagittal CT-angiography can be useful in diagnosing stylo-carotid syndrome.

Differential diagnostic considerations for Eagle’s syndrome are numerous cranio-facio-cervical pain syndromes^1,4^, e.g. neuralgias of the glossopharyngeal nerve, trigeminal nerve^1^ and pterygopalatine ganglion^9^, temporomandibular disorders^9,53^, dental problems^1,9^, cervical arthropathies or pharyngeal infections and tumors^1^.

Therapy consists of conservative management with anti-inflammatory drugs and analgesics, or transoral surgical resection of the styloid apophysis^2,4,41,46^, an operation that has been performed since 1872^10^.

## Materials and Methods

### Radiology

Two-hundred eighty-four excised hyoid-larynx complexes were radiologically examined and collected in a forensic-radiological database between 2002 and 2013^54^. The database contained anonymized patient data. Approval by a medical ethical committee for this retrospective investigation in anonymized deceased patients is not required to perform this type of study in the Netherlands. It concerned a retrospective study with anonymized data from deceased persons, so written informed consent was not required. When analyzing the data none of the research team members had access to identifying information of the persons.

Age ranged from 0 to 98 years (mean: 44 years), male-female ratio was 1:1. Radiological (X-ray) examination of the excised complexes started in the early 2000s with the use of a mammograph in the following eight directions: *AP*, *left lateral*, *right lateral cranio-caudal*, *left oblique 30* and *60 degrees*, *right oblique 30* and *60 degrees*. A few years later this examination in eight directions was replaced by a digital bucky, supplemented with a CT scan of the excised complex. This combination became the gold standard. In later phases of the study a whole-body CT was often performed before autopsy, in addition to radiological examination of the excised complex with bucky and CT. The complex could then be virtually extracted from the whole-body CT dataset: see de Bakker *et al*. (2016) for protocols^54^. An independent researcher together with an experienced radiologist scored all radiological cases for deviations from standard anatomy as shown in Fig. 1.

### Vrolik specimens

In addition to the radiological cases, we studied some profound cases with anatomical variants from the anatomical museum of the Amsterdam UMC of the University of Amsterdam, *Museum Vrolik*^55^. Images of these cases served as illustration of rare variants.

## Results

Radiological examination of the 284 excised hyoid-larynx complexes showed that only 37% met the anatomical standard (Fig. 1). A remarkable 63% of the 284 cases showed various degrees of anatomical variants. Two variations were most observed: the age-dependent uni- or bilateral ankylosis of the hyoid body with the greater horns (n = 33 and 70 respectively) and uni- or bilateral presence of triticeal cartilages in the lateral thyrohyoid ligament (n = 11 and 12 respectively) (Table 2). These minor variations do not have clinical implications. Nineteen percent of this sample of 284 excised complexes, however, portrayed relevant anatomical variants (Table 2, last column).

**Table 2:** Overview of hyoidal and stylohyoidal variations found in 284 forensic radiological hyoid-larynx scans.

Variation	Panel figure. 2	# Cases	Percentage of 284	Corrected %*
**Hyoid body**				
Bilateral ankylosis body with greater horns	a2/a3	70	24.6	
Unilateral ankylosis body with greater horns	a4/a5	33	11.6	
Unilateral ankylosis greater and lesser horn	a6	2	0.7	0.7
Exostosis median process	a7	7	2.5	2.5
**Subtotal**		**112**	**39**.**4**	**3**.**2**
**Hyoid greater horn**				
Hypoplastic on one side	a2	1	0.4	0.4
Intermittent, ankylosis body and greater horn	a3	1	0.4	0.4
Exostosis	b4	1	0.4	0.4
Curved upwards	b5	2	0.7	0.7
Curved downward	b6	1	0.4	0.4
Accessory bone	b7	3	1.1	1.1
Articulates with superior horn	d6	1	0.4	0.4
Articulates with triticeal and superior horn	d7	1	0.4	0.4
**Subtotal**		**11**	**3**.**9**	**3**.**9**
**Hyoid lesser horn**				
Unilateral absence	c2	9	3.2	3.2
Bilateral absence	c3	7	2.5	2.5
Hypoplastic on both sides	c4	1	0.4	0.4
Unilateral hyperplastic	c5	3	1.1	1.1
Bilateral hyperplastic	c6	3	1.1	1.1
Asymmetrical hyperplastic	c7	4	1.4	1.4
**Subtotal**		**27**	**9**.**5**	**9**.**5**
**Thyroid superior horn**				
Unilateral triticeal cartilage	d2	11	3.9	
Bilateral triticeal cartilage	d2	12	4.2	
Non fusion between superior horn and thyroid	d3	2	0.7	0.7
Unilateral hypoplastic	d4	1	0.4	0.4
Unilateral absence	d5	1	0.4	0.4
**Subtotal**		**27**	**9**.**5**	**1**.**4**
**Stylohyoid complex**				
Keratohyal bone	e3	1	0.4	0.4
Fundamental type	e4	1	0.4	0.4
**Subtotal**		**2**	**0**.**7**	**0**.**7**
**Total**			**63**.**0**	**18**.**7**

^*^The corrected percentage of cases comprises only the relevant anatomical variants, without the uni- or bilateral ankyloses of the greater horns and the uni- or bilateral presence of triticeal cartilages that do not have clinical implications.

All variations found in this study (Table 2) supplemented with rare cases described in literature and from *Museum Vrolik* were summarized in Fig. 2, in an attempt to provide an overview of all currently known deviations from normal anatomy of the hyoid-larynx complex. Moreover, 27 out of 33 cases with unilateral fusion between hyoid body and greater horn from which laterality and sex were known, were tabulated in Table 3. Right sided fusion was observed more frequently (n = 17) than left sided fusion (n = 10) in both sexes.

**Table 3:** Number of cases with left or right sided fusion of the hyoid body with the greater horn.

	Male	Female	Total
Left sided fusion	8	2	10
Right sided fusion	12	5	17
Total	20	7	27

## Discussion

Two thirds of the examined hyoid-larynx complexes deviated from the anatomical standard (Fig. 1). This was mostly due to minor variations like age-dependent ankylosis of the hyoid and presence of triticeal cartilages. Almost one fifth of the cases comprised more striking anatomical variants. Some of these variants have not been described in literature before. Note, however, that this sample of 284 cases may be a biased sample, because it is based on the suspicion of the pathologist of a fracture in the hyoid-larynx complex in a forensic context. Also, we examined explanted complexes and therefore elongation of the styloid process could not be determined.

### Embryonic etiology of anatomical variants

**Figure 3:**
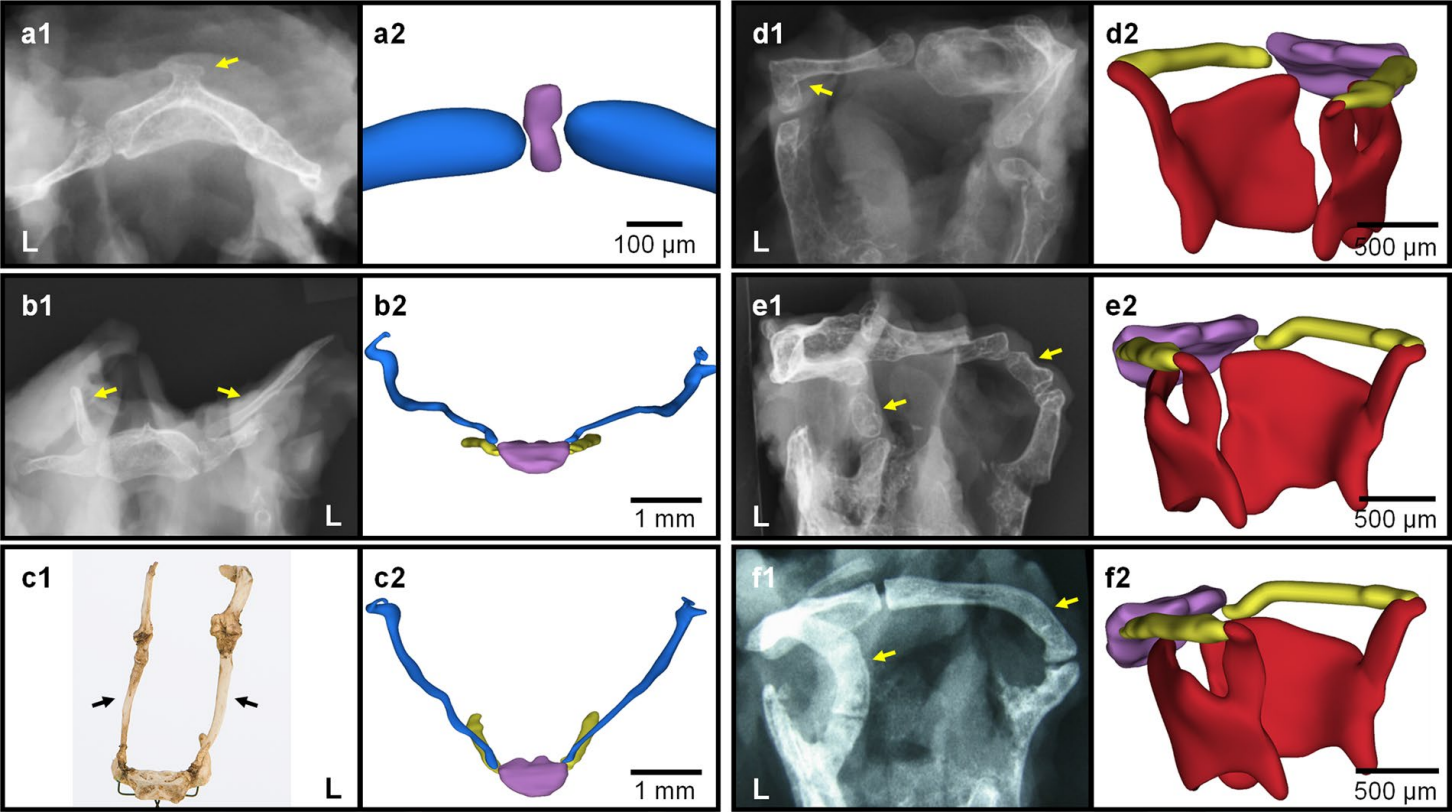
Anatomical variants of hyoid explained by embryological development. Overview of six of the most profound anatomical variants (**a1**,**b1**,**c1**,**d1**,**e1**,**f1**), compared with the embryonic development of that part of the hyoid-larynx complex (**a2**,**b2**,**c2**,**d2**,**e2**,**f2**)^56^. The hyoid bone (anlage) is shown in purple, second pharyngeal arch cartilage derivatives are shown in blue, third pharyngeal arch cartilage derivatives are shown in yellow and the thyroid cartilages are shown in red. The ‘L’ indicates the left side of the patient. Each arrow indicates the location of the variant. The shown variants are: conventional radiograph of an exostosis of the hyoid body (**a1**)(Fig. 2a7), conventional radiograph of an elongation of both lesser horns (**b1**)(Fig. 2c7), dried specimen of an ossified hyoid apparatus; the fundamental type (**c1**)* (Fig. 2e4), conventional radiograph of the left greater horn curved downward (**d1**)(Fig. 2b6), conventional radiograph of a bony connection between the greater and the superior horn, i.e. the congenital hypothyroid bar (**e1**,**f1**) (Fig. 2d7,d6). *On display in Museum Vrolik. Collection Louis Bolk, 1912. Photo by Sanne Mos & Marco de Marco; courtesy of Museum Vrolik, Amsterdam UMC, University of Amsterdam. With permission.

When comparing the most profound deviant cases with our 3D reconstructions of embryonic development^56^ (freely available at http://www.3datlasofhumanembryology.com), we noted how their etiology could be explained by embryonic development (Fig. 3).

#### The median process

A median process of the hyoid body (n = 7) can be explained by the body’s embryological origin (Fig. 3a). The hyoid bone anlage^33,57^, a cylindrical shaped growth center ventrally positioned along the cranio-caudal axis in-between the left and right-sided bar of the second pharyngeal arch cartilage, marks the first appearance of the hyoid body^56^. We hypothesize that the median process is a remnant of this cylindrical shaped anlage.

#### Second pharyngeal arch cartilage anomalies

Elongation of lesser horns (Fig. 3b), styloid process (Fig. 3e2) and ossification of the hyoid apparatus (Fig. 3c) can all be explained by a degree of stylohyoid ligament ossification. The second pharyngeal arch cartilage persisted as cartilaginous bar in this trajectory after which it ossified partially (Fig. 3b1,e2) or completely (Fig. 3c1). Up until now, Eagle’s syndrome (Fig. 2e2) and the aberrant hyoid apparatus (Fig. 2e3-7) are traditionally discussed in literature as two separate entities, leading to much confusion concerning definitions and clinical presentation. Considering the embryonic etiology of these syndromes, we propose that they are merely two expressions of a broad spectrum, all due to the partial or complete persistence of the second pharyngeal arch cartilage. These anomalies are all found in the trajectory between the lesser horn of the hyoid and the styloid process. Therefore, they should be considered as one entity, preferably referred to as ‘second pharyngeal arch cartilage anomalies’.

#### Third pharyngeal arch cartilage anomalies (congenital hyothyroid bar)

The 3D reconstructions of the embryological development of the hyoid-larynx complex show a clear connection between the dorsal part of the bars of the third pharyngeal arch cartilages and the superior horns of the thyroid, derived from condensed mesenchymal tissue. This profound connection will become the lateral thyrohyoid ligament. Fig. 3d1,e1,f1 all show various degrees of a persisting connection between greater and superior horns. The most common variation in this trajectory is presence of a triticeal cartilage (n = 11 unilateral and n = 12 bilateral). In Fig. 3d1 the left greater horn describes a 90 degree angle pointing caudal towards superior horn. In Fig. 3e1 an accessory bone articulates between the elongated superior and greater horns. In Fig. 3f1 the greater horn articulates directly with the ossified superior horn. These cases can be explained by ossification of persisting embryological cartilaginous components in the trajectory of the lateral thyrohyoid ligament and is therefore referred to as *congenital hyothyroid bar*^23^.

One case of duplication of the greater horns has been reported^5^. The hyoid would miss the lesser horns and shows a duplication of the greater horns. We suggest that these duplicated horns, in fact, are more likely to be elongated lesser horns (Fig. 2c6–7).

### Clinical relevance

Symptoms of variants in the hyoid-larynx complex are often not recognized by clinicians^34^. Great care should be taken in situations of tracheal intubation in these patients, because of the risk of regurgitation and aspiration^58–60^. Anomalies of this complex are of great importance for radiological examination and surgery of the neck region, but they should also be known by forensic experts, anthropologists, anatomists and dentists^1,5^ to appreciate the clinical impact of these variants and to avoid judicial errors in cases of assumed strangulation or blunt neck trauma.

### Forensic relevance

Fractures in the hyoid-larynx complex are one of the best indicators of strangulation^5–8^, but they can also be caused by for instance hanging, traffic accidents, osteoporosis senilis, sporting accidents and after intubation^8,14,20,24,42,61–64^. Due to the considerable frequency of anatomical variations of the hyoid-larynx complex, great care should be taken when diagnosing traumatic lesions of this complex^5,8,12^.

In the normal process of ageing, the triticeal cartilage (Fig. 2d2) in the lateral thyrohyoid ligament often calcifies, which enables radiographic detection. However, when this cartilage is intensely and inhomogeneous calcified, it should not be mistaken for an avulsed fracture of the upper horn of the thyroid cartilage^23,65^. Therefore, oblique radiographs should be used for further radiological examination, to distinguish a fracture from an inhomogeneously calcified cartilage^12^.

Most fractures are found in the upper thyroid horns^8^. Fracturing of the hyoid occurs mainly between greater horns and body, when ankylosis is incomplete, or in the posterior part of the greater horn^8,14,63,66^. Since ankylosis of the hyoid joints is age dependent, fractures occur more frequent in persons aged above 30^5,8,13,14,18,20–24,67^. D’Souza even stated that when a victim is over 38 years, clinicians and forensic experts can expect a fractured hyoid, after pressure on the neck^20^. Joint luxation between hyoid body and greater horn has also been reported in cases of strangulation^63^.

Some anatomical variants resemble fractures. All examples in Fig. 2 should be kept in mind when the hyoid-larynx complex is examined during medico-legal examination but especially examples b3, b7, d2, d3, d6 and d7 should always be considered when a suspected fracture is found in those locations. When the difference between variant and fracture remains ambiguous after autopsy and radiological examination, histological examination of the affected part should be performed.

### Ethical approval

For this type of study formal consent is not required. This article does not contain any studies with animals performed by any of the authors.

## Conclusion

We provided an overview of the known anatomical variants of the hyoid-larynx complex, with relevance for clinicians and forensic experts. Etiology of the variants has been declared by their development. Since the aberrant hyoid apparatus and Eagle’s syndrome are often intermingled in literature as they are both explained by persistence of second pharyngeal arch cartilage, we propose to refer to them as ‘second pharyngeal arch cartilage anomalies’.
